# Failure to thrive in infant and toddlers: a practical flowchart-based approach in a hospital setting

**DOI:** 10.1186/s13052-021-01017-4

**Published:** 2021-03-10

**Authors:** Roberto Franceschi, Caterina Rizzardi, Evelina Maines, Alice Liguori, Massimo Soffiati, Gianluca Tornese

**Affiliations:** 1 Division of Pediatrics, S. Chiara General Hospital, Largo Medaglie d’Oro, 9, 38122 Trento, Italy; 2 Institute for Maternal and Child Health, IRCCS Burlo Garofolo, Trieste, Italy

**Keywords:** Failure to thrive, Flow chart, Hospital setting

## Abstract

**Background:**

Failure to thrive is a common reason for referral to paediatric services. Malnutrition or inadequate caloric intake is the most common cause, while organic form is unlikely in children who are asymptomatic and healthy on examination. By this study we evaluate the application of a cost-effective flow chart that helps the clinician in a hospital setting discern accurately organic and non-organic failure to thrive.

**Methods:**

Conduct a prospective single-center study in children up to 2 years of age with growth faltering. The pediatricians used a practical flow chart, took the medical history, created a growth chart, performed clinical examinations, and requested blood test and consultations in a step by step approach.

**Results:**

Among the 74 subjects included in the study, the diagnosis of organic failure to thrive was reached by 42%. Gastrointestinal and genetic diagnoses were the most frequent. Patients with organic failure to thrive had significantly lower gestational age and birth weight. Age at diagnosis and Z-score weight were lower in organic than in non-organic forms. Most patients with non-organic forms (88%) did not undergo in-depth blood test or specialist advice.

**Conclusion:**

The flow chart we presented was accurate for diagnosing children with failure to thrive in a hospital setting and distinct organic and non-organic forms. It was cost-effective to avoid unnecessary blood test or consultations in most non-organic diagnoses.

## Background

The term “failure to thrive” (FTT) is often used for infants and children with weight below the 5th percentile according to gender; and age; however, there is no objective consensus on its definition. Supporting definitions includes weight for length below the 5th percentile; body mass index for age below the 5th percentile; or a sustained drop in growth velocity, in which weight for age or weight for length/height decreases by two major percentiles (percentile markers 95th, 90th, 75th, 50th, 25th, 10th, and 5th) over time [1–6]*.* It appears mainly in 1–2-year-old children but can occur at any time during childhood [4].

Although problems in achieving or maintaining appropriate weight are the predominant manifestations of FTT, ongoing severe malnutrition impairs overall growth, affecting weight, head length, circumference and, in extreme cases, can compromise the development of cognitive abilities and appropriate immune function, failing to achieve developmental milestones and normal health [6].

Many factors have been reported to be associated with the condition, including biological factors such as parental measurement, social factors including deprivation, maternal educational level, family size, and a wide range of physical conditions. Malnutrition or inadequate caloric intake is the most common cause of FTT. Organic FTT (OFTT) is unlikely in children who are asymptomatic and healthy on examination [3, 7].

The family doctor who works closely with the community is in an optimal position to detect FTT in children when they present with illnesses or for a healthy balance. Primary care can document the type and amount of food the child consumes and observe subsequent consistent weight gain in 1–2 weeks, confirming the diagnosis of non-organic FTT (NOFTT). So far, there is still no evidence to support the extensive, systematic use of screening laboratory evaluations in FTT: investigations should be performed in the presence of signs or symptoms of the disease or where weight loss is persistent or severe [6]. Inpatient monitoring is not advisable, except in very extreme circumstances. Infants need only be referred to a secondary care pediatrician in case of [6, 8]:

- extreme parental impairment or anxiety;
- extremely poor parent-child interaction;
- need for precise documentation of nutritional intake;
- psychosocial factors that put the child’s safety at risk;
- underlying severe illness or medical problem or features that suggest an associated disease;
- severe malnutrition or dehydration or severe weight faltering (a drop of two or more centile spaces on the WHO chart) persisted despite the community and dietetic interventions.

Hospitalization allows the clinicians to exclude organic disease by expediting and efficiently analyzing laboratory studies, radiologic examinations, and specialist consultations. In some patients with severe inanition, nutritional supplementation by nasogastric tube feeding or gastrostomy feeding can be adopted if a long satiety period is predicted [9].

A semi-objective diagnosis tool for identifying which patients may benefit from further evaluation and interventions to optimize growth and development has been published previously [10].

## Methods

### Aim and setting

We propose a flow chart suggesting diagnostic possibilities according to history, growth chart, and clinical evaluation to rule out possible pathology based on a rational, cost-efficient, case-based approach.

This study is single-center prospective research conducted in the Department of Pediatrics at the S. Chiara Hospital in Trento (Italy), a secondary level center, from January 2015 to December 2019.

### Partecipants

This study involved all children within 2 years of age with faltering growth. Patients were enrolled after the initial consultation in the pediatric gastroenterology, endocrinology clinics, or after evaluation in the pediatric emergency room for severe malnutrition or dehydration.

Growth faltering was defined as weight deceleration higher than 1 Z score in the previous 6 months or the descending crossing of more than two major percentiles (95th, 90th, 75th, 50th, 25th, 10th, and 5th). Premature infants (< 37 gestational weeks) were included, and corrected gestation age was used up to age 2. The exclusion criteria were: children > 2 years; children with known organic etiology of their FTT (i.e., subjects with celiac disease, hypothyroidism, cardiac defect).

### Design

Growth assessment of weight, height, supine length and head circumference was performed at the pediatric clinic’s admission by the nursing team previously trained on measurement techniques. We used WHO growth charts for the weight (WT), length (LT), and head circumference (HC). The weight/length ratio was plotted on the 2006 reference curves of the World Health Organization (WHO) [11].

**Fig. 1:**
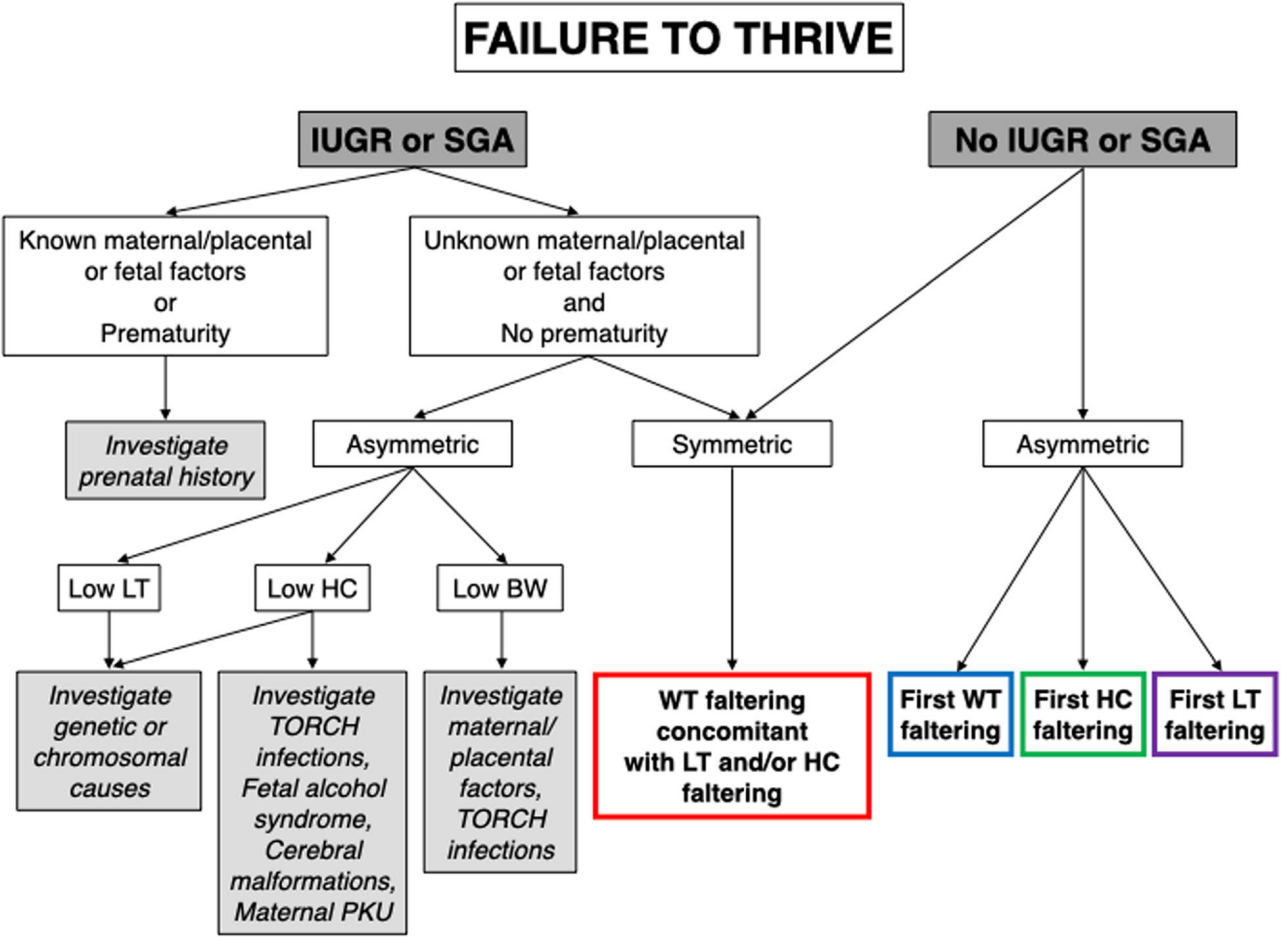
failure to thrive starting approach. IUGR: intrauterine growth restriction. SGA: small for gestational age. WT: weight, LT: length, HC: head circumference. BW: birth weight

**Fig. 2:**
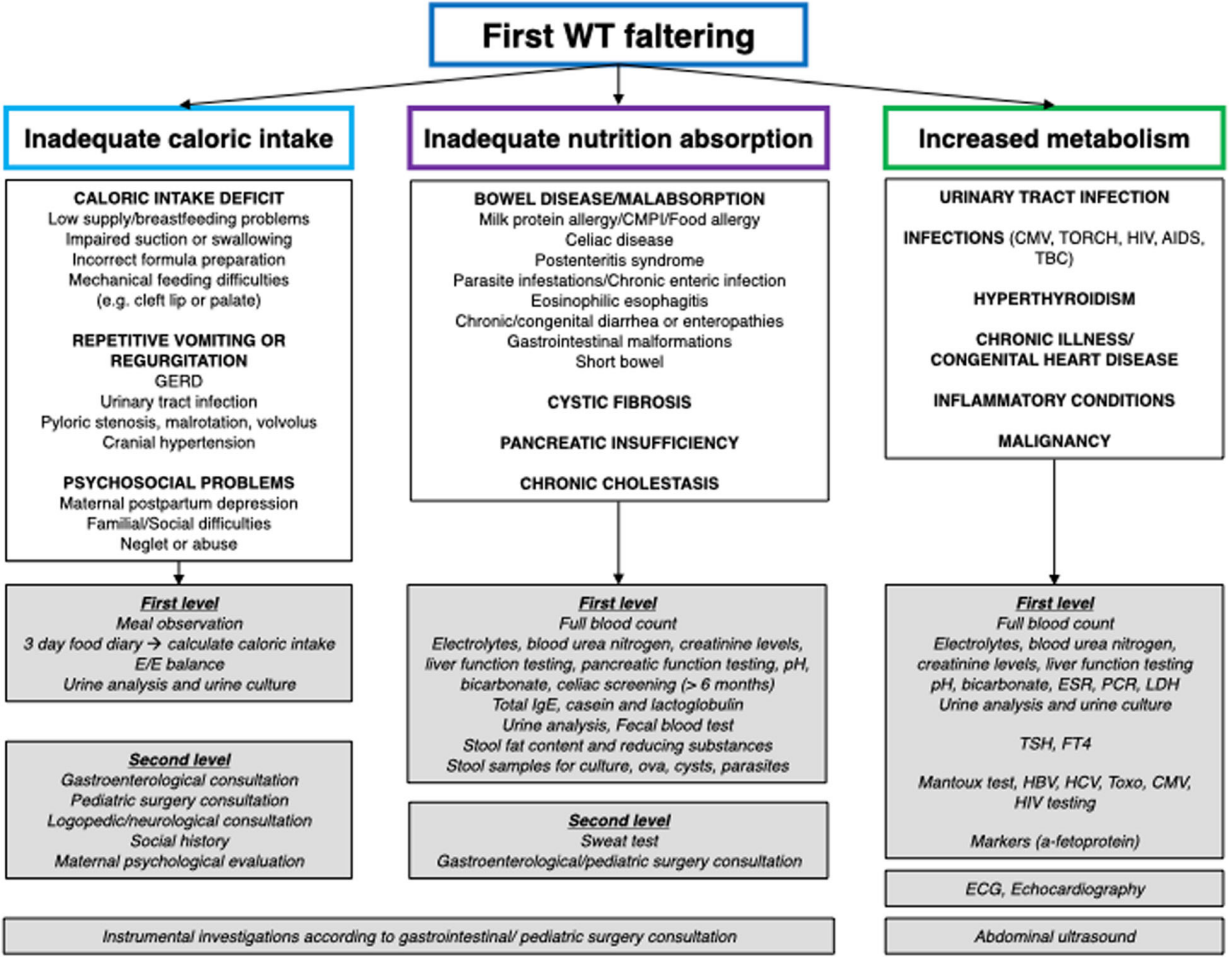
approach to first weight (WT) faltering. GERD: gastroesophageal reflux disease. E/E: entry/exit balance. CMPI: intolerance to cow’s milk proteins

**Fig. 3:**
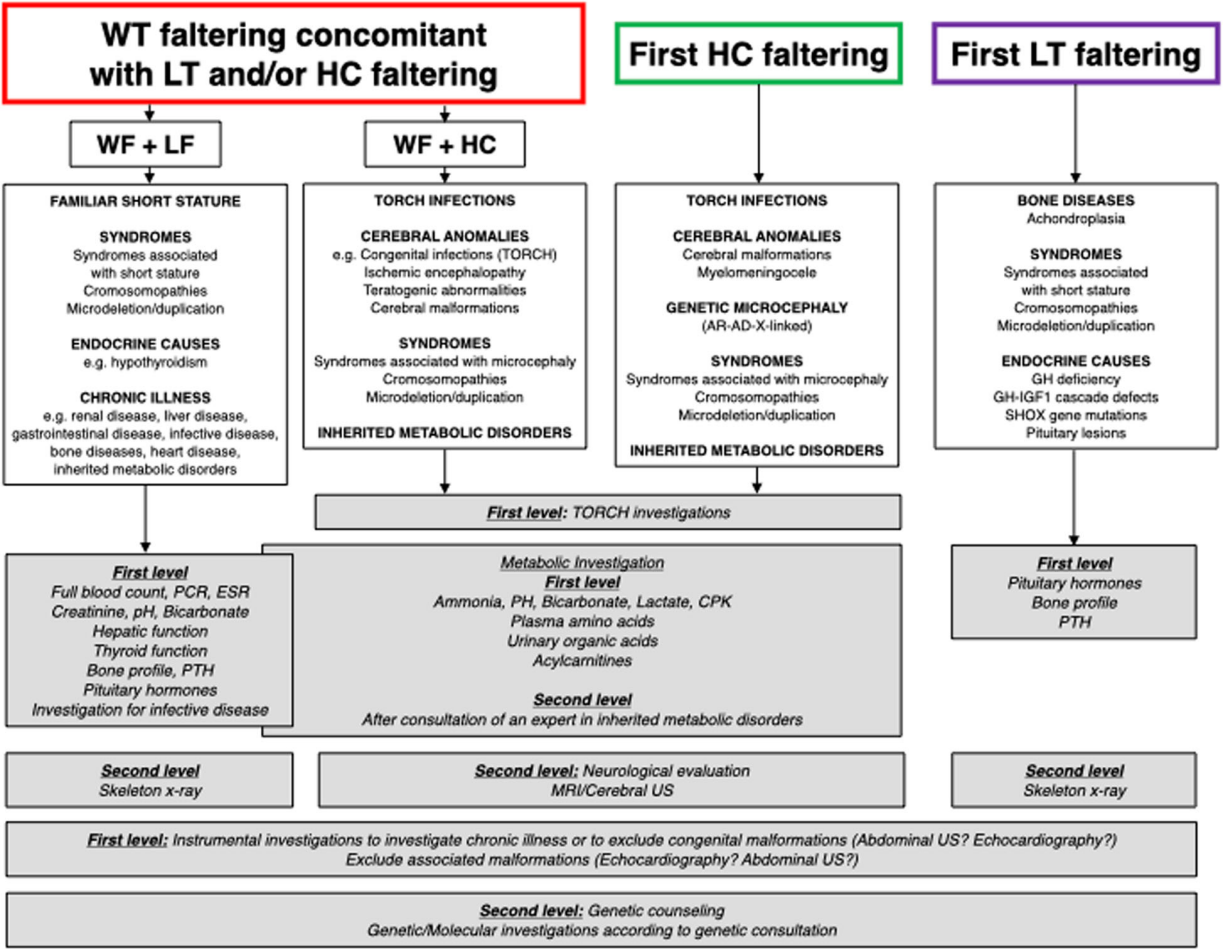
approach to weight (WT) faltering concomitant with length (LT) and/or head circumference (HC) faltering; approach to first HC faltering or first LT faltering

Pediatricians in the hospital used a hands-on flow chart (Figs. 1, 2, 3), and conducted the history, created a growth chart, performed clinical exams, and requested blood tests and consultations with a step-by-step approach. The flow chart employed was developed based on the literature review and included criteria to suggest diagnostic possibilities in FTT at primary and secondary care level.. Timing of faltering growth onset (prenatal/postnatal) and subsequently distinction of growth failure in symmetric (involving weight, length and head circumference) or asymmetric (involving only one or two parameters) are the starting points (Fig. 1).

Subsequently clinician follows flow chart reported in Fig. 2 if the baby is first WT faltering, excluding at first inadequate caloric intake on the basis of medical history, clinical examination, food diary and entry/exit balance. If caloric intake is adeguate, inadeguate absorption or increased metabolism has to be excluded by medical history, clinical examination and blood tests.

Patients, 2–4 weeks after discharge, were referred to specialist consultants in the case of OFTT or family pediatricians in the case of NOFTT, to verify a recovery of growth.

Analyzes were performed using SPSS Software (SPSS, Inc.), and data were expressed in descriptive statistics. Shapiro test has found that WT, LT and HC follow a normal distribution. The Independent t-test was used to compare the means of two independent groups, while the Chi-squared test was employed to examine medians of two independent groups. Statistical significance was recognized when *p* < 0.05.

## Results

We have admitted 3127 patients in 5 years to our Pediatric Department. During this period, we enrolled 82 patients who met the inclusion criteria for our study. Due to incomplete anthropometric data from clinical notes, 8 patients were not included in the analysis.

Among the 74 subjects included in the study, 42 were male and 32 females, 61 were of caucasian origin, 8 Afro-American, 5 Asian. 4 children (5%) were born SGA and 3 children (4%) were premature. The mean age was 6.98 ± 2.45 months.

**Fig. 4:**
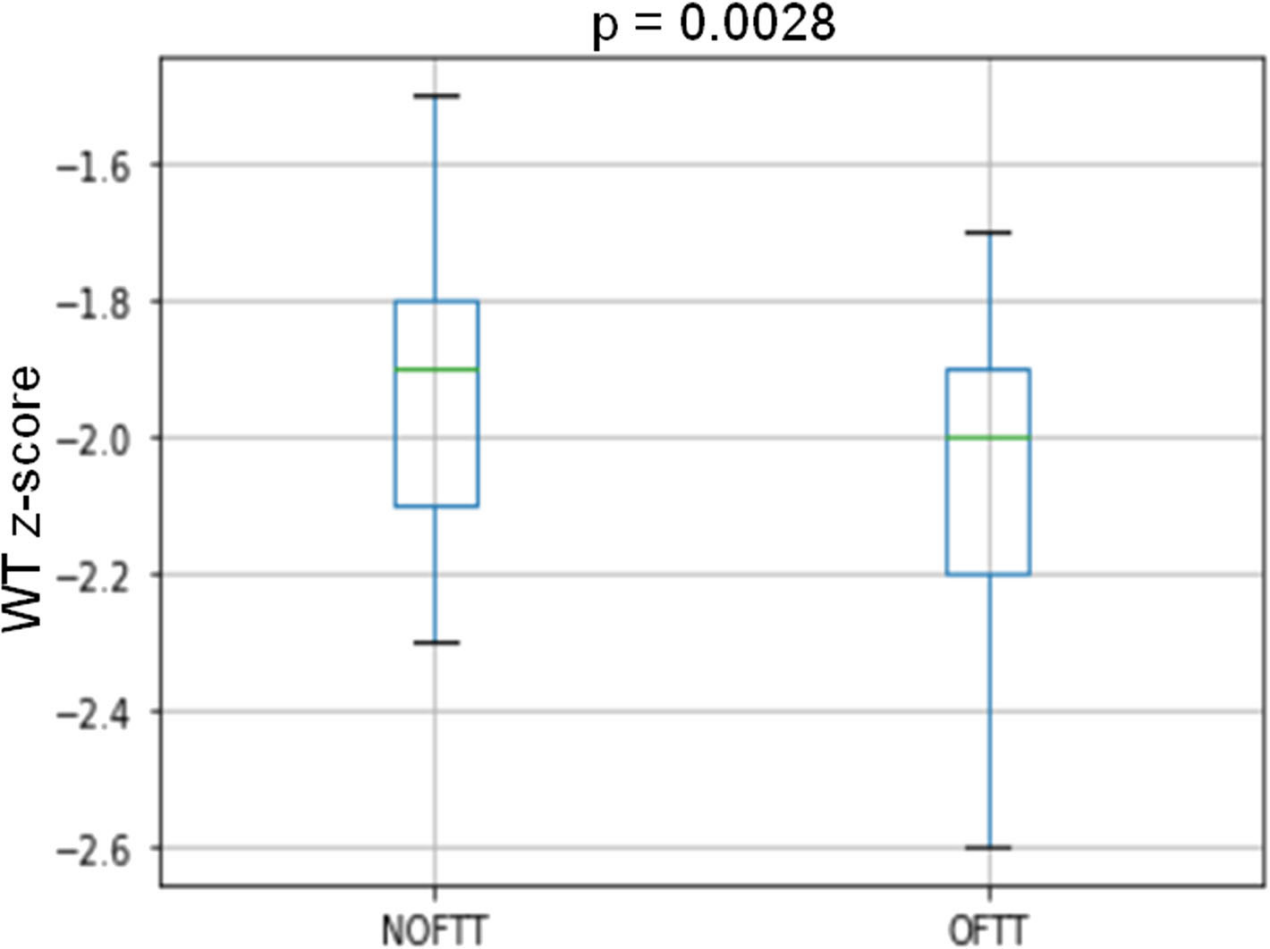
box plot of weight (WT) z-score in non-organic failure to thrive (NOFTT) and organic failure to thrive (OFTT). The box’s centerline denotes the median value, the box’s extremes, the interquartile range, and the bars, the upper and lower limits of 95% of the data, *p* value by chi-square test

The main reasons for admissions were to obtain precise documentation of nutritional intake, severe malnutrition, dehydration, and suspicion of OFTT (Table 1). Nutritional repletion by nasogastric tube feeding was adopted in 9 patients. Using flow charts, hospital pediatricians reached the diagnose of OFTT in 31 patients (42%) and NOFTT in 43 subjects. Gastrointestinal (n°12) and genetic (n°8) diagnosis were the most common (Table 2). Patients with OFTT had significantly lower gestational age and birth weight than NOFTT subjects (Table 3). Age at the diagnosis and weight Z-score were lower in OFTT compared to NOFTT (Table 3, Fig. 4). Length and head circumference were not significantly different in the two groups, but the length z-score showed a tendency to be lower in OFTT. Weight for length was lower in NOFTT, but the difference was not statistically significant (Table 3). According to our flow chart, most of the NOFTT subjects (n° 34/43; 88%) did not perform a receive in-depth blood test or specialist consultation. 8/34 patients performed full blood count, creatinine, liver function and electrolytes at the emergency room; our pediatric nurse’s food diary and caregivers’ behavior monitor were enough to formulate a diagnosis. The family pediatrician followed up subjects with NOFTT, and we did not register any repeated hospital admission by these patients in 5-year time. The subjects with OFTT were followed up in our hospital’s specialist clinic or tertiary care centers.

**Table 1:** Reasons for hospitalizations in patients with failure to thrive

Reasons for hospitalization	N
The need for precise documentation of nutritional intake	14
Severe malnutrition and/or dehydration (> 10%)	22
Suspicion of severe underlying illness based on critical weight faltering that has persisted despite community and dietetic interventions	12
To exclude organic disease through s quick and efficient panel of laboratory studies, radiologic examinations, and specialist consultations	15
Suspicion of neglect	3
Extreme parental impairment or anxiety	5
Not reported in the clinical note	3
**Total**	74

**Table 2:** Final diagnosis in the 74 patients admitted for Failure To Thrive (FTT)

Final diagnosis	N
**Non-Organic Failure To Thrive (NOFTT)**	
Caloric intake deficit	38
Preceding infection	5
**Total**	43
**Organic Failure To Thrive (OFTT)**	
Gastrointestinal problems: GERD [4], malrotation [1], CMPI [4], biliary atresia [1], hepatic insufficiency [1], eosinophilic esophagitis [1]	12
Genetic conditions: Turner syndrome [1], Noonan syndrome [1], Silver Russel syndrome [2], Prader Willi syndrome [2], other genetic syndromes [2]	8
Metabolic condition: fatty acid oxidation defect [1], congenital disorder of glycosylation [1]	2
Cardiac disease: aortic coarctation [1], large defect of the interventricular septum [1]	2
Renal tubular acidosis	1
Malignancy	1
CMV congenital infection	1
Fetal alcohol syndrome	1
SOD	1
Central hypothyroidism	1
Achondroplasia	1
**Total**	31

Abbreviations

*GERD Gastroesophageal reflux disease. CMPI* intolerance to cow’s milk proteins. *SOD* septo optic dysplasia

**Table 3:** characteristics of infants with growth faltering at the first visit to the hospital

	All (n°74)	NOFTT (n°43)	OFTT (n°31)	*p*-value
Sex (M/F)	42/32	23/20	19/12	0.37
Gestational age (week)	38.8 ± 1.15	39.16 ± 0.97	38.3 ± 1.2	**0.0027**
Birth WT (Kg)	2.90 ± 0.79	2.99 ± 0.23	2.76 ± 0.37	**0.0018**
Age at first visit (months)	6.98 ± 2.45	8.01 ± 2.15	5.54 ± 2.12	**< 0.0001**
WT z-score	−2.0 ± 0.23	−1.93 ± 0.39	−2.09 ± 0.25	**0.0028**
LT z-score	−0.94 ± 0.79	− 0.89 ± 0.73	− 0.99 ± 0.82	0.59
HC z-score	−0.26 ± 0.90	− 0.12 ± 0.76	− 0.47 ± 1.04	0.10
Weight for length ratio	−2.3 ± 1.02	−2.4 ± 0.95	− 2.1 ± 1.11	0.186

*M* male, *F* female. *WT* weight, *LT* length, *HC* head circumference. Data are reported as mean ± SD

## Discussion

The prevalence of FTT depends on the population studied and recognition criteria used. In the United States, FTT can occur in up to 10% of children in primary care and approximately 5% of admitted children [4, 12]. In our cohort, FTT patients were 2.6% (82 out of 3127) of subjects who were hospitalized in 5 years. The rate of detection also depends on the vigilance of individual physicians [13]. Early studies of FTT were hospital-based, but in recent years structured ambulatory management has been recognized as more cost-effective [14], more acceptable to patients and their families, and more likely to succeed [15]. In our clinical practice, the family pediatrician primary care approach is the gold standard for patients with FTT and gastrointestinal or endocrine consultations are filters for possible hospitalization. The most common reasons for hospitalization of patients with FTT in our department, as reported in this study, were clinical deterioration (severe malnutrition and dehydration) or the need to confirm the suspicion of NOFTT by precise nutritional intake’s documentation, or to exclude OFTT.

Major organic causes of FTT are rare. UK population-based studies found the organic disease in only 5–10% of children with slow weight gain [16, 17], and all the subjects presented with symptoms or signs suggesting underlying disease. Regarding 2 US hospital studies of children with FTT, in the first (in 1978), Sills et al. retrospectively assessed 185 hospitalized patients for FTT evaluation: 18% had proven organic etiologies, strongly suggested by the history and physical examination in all these patients; 50% failed to thrive due to environmental deprivation; only 1.4% of the laboratory studies performed were helpful in reaching a diagnosis [18]. In the second (in 1981), Homer et al. performed a retrospective chart analysis of 82 hospitalized children: 21 cases (26%) had organic causes, 34 had nonorganic causes, and 19 had both organic and nonorganic causes [19]. In a Korean study, 123 patients admitted during their first two years of life received an FTT diagnosis, 80 cases (65.0%) were NOFTT. Weight decline was very severe in organic FTT patients and younger patients at the first visit [4].

In our hospital-based study, with patients admitted to the pediatric ward after specialist evaluation or from the emergency room, after using diagnostic flow charts, we concluded with a diagnosis of OFTT in 31/74 (42%) subjects, similar to the Korean study, which is the most recent published according to our knowledge. Instead, this prevalence is higher than the one detected in previous studies performed in the nineties, when structured outpatient management and economic strategies were probably less applied.

In our cohort, patients with gastrointestinal and genetic diseases were the most represented in the OFTT group, but we cannot conclude that these are the leading causes of OFTT because hospitalized children with a known organic etiology of their FTT were not enrolled in this study. A high prevalence of gastrointestinal disorders has already been reported in other cohorts [4, 20]; the high prevalence of children with genetic conditions in our cohort has never been reported in previous studies performed mainly in general pediatrics or gastroenterology centers, while in this study there are also referrals from endocrinology centers that can intercept genetic condition related to short stature.

The age at first visit for FTT in our cohort (6.98 ± 2.45 months) was lower than that reported in the literature [4, 16], probably due to the high prevalence of OFTT. Indeed, patients with OFTT presented a more severe phenotype than NOFTT: at birth, they had lower gestational age and birth weight and in the postnatal period they presented with earlier onset of weight faltering, weight more compromised and also a trend to worse length parameters. These data are confirmed by other studies [4].

This study presents the limit that we do not really know the sensitivity and specificity of the flow chart, since the children were not undergoing the same investigations for ethical reasons. However it is reasonable that the flow chart was able to pick up the right children, diagnosing them into the different categories “organic” and “non-organic”, because after diagnosis they recovered in growth and we did not register any repeated hospital admission.

## Conclusions

In this investigation, we presented the application of a cost-effective flow chart that helped the clinicians to accurately diagnose children with FTT in the hospital setting by discerning a high prevalence of OFTT (42%) who had a more severe phenotype, and NOFTT, which did not receive unnecessary in-depth blood tests or consultations in 88% of cases.

## Data Availability

The datasets used and analysed during the current study are available from the corresponding author on reasonable request.

## Abbreviations

FTT: Failure to thrive
NOFTT: Non-organic failure to thrive
OFTT: Organic failure to thrive
IUGR: Intrauterine growth restriction
SGA: Small for gestational age
WT: Weight
LT: Length
HC: Head circumference
BW: Birth weight
